# Self-Adaptive Learning of Task Offloading in Mobile Edge Computing Systems

**DOI:** 10.3390/e23091146

**Published:** 2021-08-31

**Authors:** Peng Huang, Minjiang Deng, Zhiliang Kang, Qinshan Liu, Lijia Xu

**Affiliations:** College of Mechanical and Electrical Engineering, Sichuan Agricultural University, Ya’an 625000, China; hpsjdyd@sicau.edu.cn (P.H.); 2019217013@stu.sicau.edu.cn (M.D.); 12200@sicau.edu.cn (Z.K.); 2019317018@stu.sicau.edu.cn (Q.L.)

**Keywords:** MEC, resource allocation, task offloading, self-adaptive learning

## Abstract

Mobile edge computing (MEC) focuses on transferring computing resources close to the user’s device, and it provides high-performance and low-delay services for mobile devices. It is an effective method to deal with computationally intensive and delay-sensitive tasks. Given the large number of underutilized computing resources for mobile devices in urban areas, leveraging these underutilized resources offers tremendous opportunities and value. Considering the spatiotemporal dynamics of user devices, the uncertainty of rich computing resources and the state of network channels in the MEC system, computing resource allocation in mobile devices with idle computing resources will affect the response time of task requesting. To solve these problems, this paper considers the case in which a mobile device can learn from a neighboring IoT device when offloading a computing request. On this basis, a novel self-adaptive learning of task offloading algorithm (SAda) is designed to minimize the average offloading delay in the MEC system. SAda adopts a distributed working mode and has a perception function to adapt to the dynamic environment in reality; it does not require frequent access to equipment information. Extensive simulations demonstrate that SAda achieves preferable latency performance and low learning error compared to the existing upper bound algorithms.

## 1. Introduction

Since the birth of cloud computing, it has changed the methods of data acquisition, storage and processing to a large extent. It involves the integration and utilization of the Internet, mobile network and cable TV network technology, making it a highly integrated network. There are also many emerging businesses, such as video conferencing, telemedicine, online games, cloud storage, financial cloud services, etc. However, due to the high complexity, high data intensity and delay sensitivity of these applications, they are limited by the device computing resources and cost. Offloading computing task requirements to the traditional cloud is one solution, but the traditional cloud is typically far away from the application, which creates a new problem—increased latency. It has been found that if some of the requests with low computational requirements and small data volume are not sent to the cloud, but processed by the server close to the data requirements, the transmission path of the request tasks will be greatly reduced. Involving the placement of small servers at the edge of the cellular network, a new distributed architecture known as mobile edge computing (MEC) has been proposed by the European Telecommunication Standard Institute (ETSI) [1]. In the MEC architecture, distributed mobile edge servers called cloudlets are deployed at the edge of wireless network to provide computing resources and storage with high bandwidth and low delay [2].

Simply offloading tasks to the cloudlets is insufficient to reduce the response latency. When cloudlets are overloaded, a large number of tasks cannot be processed in a timely manner due to limited computing resources, resulting in non-negligible computing delays. Due to the spatiotemporal dynamics of user devices, the appearance time of each user device in the communication range is random, which means that the cloudlet is not available for user devices that are not within the communication range. Meanwhile, many user devices are idle at most times, while some user devices are slow to respond to tasks due to the shortage of computing resources. Reasonable recycling of the idle computing resources of user devices and cloudlets can greatly improve the utilization of user equipment and provide a faster service response for users.

In this paper, our main contributions are as follows:

(1) We propose a self-adaptive learning of task offloading algorithm (SAda) to guide task offload and minimize task delay. The operation of the SAda algorithm is distributed and can improve the offloading performance by learning from the previous offloading experience.
(2) Considering the spatiotemporal dynamics of the user’s device, the perceptual function is also integrated into the algorithm. The user devices that require the task processing service are called the task user equipment (TUE), and the user device that can serve the TUE for the task processing is the service user equipment (SUE). The TUE can sense the position and working state of the SUE when task processing is needed, and the computation task can be reasonably assigned to a suitable SUE, which can obtain the optimal task assignment scheme and the minimum delay.
(3) A learning method during offloading tasks is introduced. With no need to obtain the exact transfer rate and CPU resources, TUEs can observe and learn during offloading computing tasks and can select candidate SUEs based on past offloading experience. In order to prove the performance of SAda, the learning errors of SAda are evaluated with three existing algorithms, which represents the difference between the delay of offloading one bit of the task during the learning process and the optimal bit offloading delay. On this basis, we carry out extensive simulations that show that the proposed method achieves good learning performance and can improve the task processing delay.

## 2. Related Works

Computing resources are moved from a remote cloud to the cloudlets, which are close to the mobile device in the MEC system. In order to improve the user experience and reduce latency, Xu et al. [3] proposed an enhanced service framework based on microservice management and a client support provider for efficient user experiments in the edge computing environment, in order to provide the edge computing service and management in the network edge. Wang et al. [4] proposed an optimization strategy for computing resource allocation in massive IoT devices considering privacy protection in a cloud edge computing environment. Since quality of service (QoS) can be affected by the choice of fog nodes and wireless transmission, Zhao et al. [5] proposed a novel data analytics framework for edge computing. The framework is based on a new decentralized algorithm, which enables all the nodes to obtain the global optimal model without sharing raw data. Liu et al. [6] attempt to keep the data of edge devices and end users in their local storage in order to eliminate any risk to user privacy, and they integrate federated learning and edge computing to propose a privacy-preserving framework that can construct a unified deep learning model across multiple users or devices without uploading their data to a centralized server. To prevent poor QoS in edge computing, Deng et al. [7] introduced a strategy with two stages during burst load evacuation. Based on an optimal routing search at the dispatching stage, tasks will be migrated from the server in which the burst load occurs to other servers as soon as possible. Guo et al. [8] proposed the mobile-assisted edge computing framework to improve the QoS of fixed edge nodes by exploiting mobile edge nodes. They devised a Credible, Reciprocal and Incentive (CRI) auction mechanism to stimulate mobile edge nodes to participate in the services for user requests. Tang et al. [9] considered a probabilistic-QoS-aware approach to multi-workflow scheduling upon edge servers and resources. It leverages a probability mass function-based QoS aggregation model and a discrete firefly algorithm to generate the multi-workflow scheduling plans.

Joint power consumption and resource management schemes have been considered to maximize overall task offload rates and long-term network stability. Yang et al. [10] comprehensively considered cloudlet placement and workload scheduling in MEC systems and forecast the future distribution of user tasks by observing user mobility habits, and service access patterns, service placement and online load scheduling can be adjusted to achieve a tradeoff between the average response time of a user request and the mobile operator costs. Ren et al. [11] analyzed the computational allocation problem in a layered network architecture, in which tasks were first received by the nearby micro-BSs and then transmitted to the macro-BS attached with MEC server after aggregation. Zheng et al. [12] have considered the variability of available resources, and the award and cost of a vehicular cloud computing (VCC) system, and proposed an optimal computing resource allocation scheme that aims to maximize the total long-term system reward. Guo et al. [13] proposed a method that was studied in a small cellular network through joint optimization of the computing offloading decision, spectrum, power consumption and computing resource allocation. Liu et al. [14] considered a method to allocate user requests in the signal coverage area of edge servers. Mazza et al. [15] developed a cluster-based computing offloading technology that aimed to minimize the cost function by integrating the balance relationship between energy consumption and task processing time.

In order to balance the workload between edge servers and minimize the access delay between users and edge servers, Jiang et al. [16] proposed an edge computing node deployment method for smart manufacturing; by comprehensively balancing the network delay and computing resources, the optimal deployment number of edge computing nodes is obtained by using an improved k-means clustering algorithm. Steffenel et al. [17] considered unbalanced networks and resource heterogeneity when optimizing data transfer and applications’ performance in the edge. They explored these two concerns through a comprehensive set of benchmarks and illustrated their importance with the help of data locality and context awareness. Xiao et al. [18] considered the multi-user and multi-server MEC system and solved it through matching theory and heuristic ideas. Mukherjee et al. [19] proposed an optimal cloudlet selection strategy under the multi-cloud environment. Chen et al. [20] have analyzed and discussed the offloading problem of multi-user computing in an MEC system. Hao et al. [21] proposed an optimal IoT service offloading (OSO) method with uncertainty that considers completion time and load balance variance optimization goals for developing offloading strategies; then, the non-dominated sorting genetic algorithm-II (NSGA-II) was fully investigated to improve the performance regarding the completion time and load balance variance. However, these existing efforts only considered the situation involving cloudlet systems with continuous connectivity, and they did not take into account the impact of mobility due to offload failure.

In order to improve the service reliability of MEC, task replication technology was introduced by Jiang et al. [22], where copies of tasks can be offloaded to multiple mobile devices for simultaneous processing. However, centralized frameworks require frequent updates of state information to optimize system performance, resulting in high signal overhead, which is a key drawback. Considering the spatiotemporal dynamics of the user devices and the signaling overhead, it is feasible for the task generator to make the task offload decision in a distributed way. Furthermore, it would be significant to design a workload offloading scheme with low computational complexity when the occurrence rate of devices is at a peak. Based on the limited processing capacity of edge nodes, a new edge computing offloading strategy has been designed to optimize the offloading experience learning performance of IoT devices. Li et al. [23] introduced a learning method to achieve high-performance workload allocation in the edge computing environment. Sun et al. [24] considered the situation of offloading computing tasks in the VEC system, in which the vehicle could learn the offloading delay experience of its neighboring vehicles when offloading computing tasks, so as to reduce the delay. However, the influence of different user devices’ moving speeds on the whole learning process and offloading process was not taken into account.

## 3. System Model and Problem Formulation

### 3.1. System Model

The devices that generate the task request are called the user equipment (UE). When there are surplus computing resources on adjacent user devices, a UE task request is offloaded to the standby server considering maximized resource utilization.

**Figure 1:**
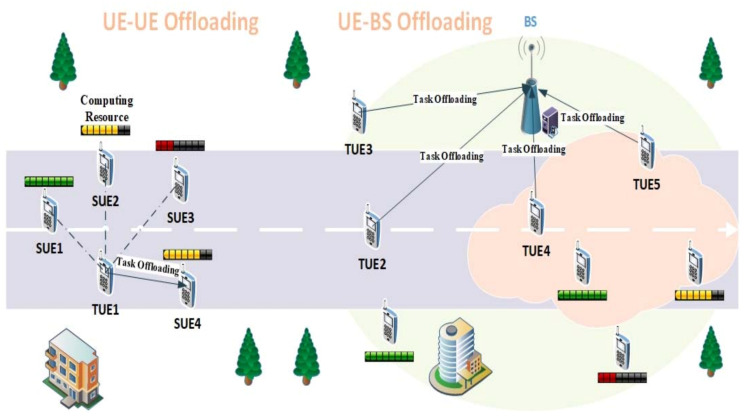
A task offloading model in MEC system.

The task offloading mechanism in the MEC system is mainly divided into the following two types [24]:

UE–UE offloading: The user devices involved in UE–UE offloading in the MEC system are divided into two categories. The TUEs are the user devices that currently produce task requests, and the SUEs are the user devices that can provide enough surplus computing resources. The functionality of each user device depends on the adequacy of its computing resources and changes over time. The TUEs can offload tasks to nearby SUEs. Each TUE may have several candidate SUEs to handle tasks, and each task can only be offloaded to a single SUE and executed. A task offloading model is shown in Figure 1: there are four candidate SUEs (SUE 1–4) for TUE1, and the current tasks are offloaded to SUE 4 because of the distance between them and the sufficient computing resources of SUE 4.

UE–BS offloading: When there are no suitable SUEs for the TUE within the communication range, or the cloudlets that are attached to the base stations (BSs) are closer to the TUEs in the communication range, TUEs will directly offload the tasks to the BSs with cloudlets for processing. As shown in Figure 1, TUE 2 and TUE 3 offload the tasks to the cloudlet as SUE, TUE 4 and TUE 5 are unsuitable for offloading tasks to the cloudlet due to distance issues.

In addition, each TUE can determine the optimal SUE independently, without internal collaboration between TUEs. We do not make any assumptions about an SUE’s conditions of service and UE mobility models. All the variables used in this paper are shown in Table 1.

### 3.2. Problem Formulation

Task offloading decision: Since the offload decision is distributed, we focus on the offload problem of the TUE model. An MEC system has three task procedures in each period, namely SUE perception, task offloading and task processing.

SUE perception: TUE selects as SUEs those UEs within its communication range that are adjacent and have the same movement direction as SUEs. The real-time status of each UE, including movement speed, position and direction of movement, can be obtained through the communication protocol. For instance, in the dedicated short-range communication (DSRC) standard [25], periodic beaconing messages can provide these status messages. This is used to represent the set of candidate SUEs within a time period T, which may change as the user’s device moves.

Given the uncertainty of each user’s device mobility, the candidate SUE set $N\left(t\right)$ is unknown for the next time period. In addition, assuming that $\forall t,\;N\left(t\right)\neq \emptyset$, the TUE can offload task requests to the edge cloud for processing.

Task offloading: After updating the candidate SUE set in each new time period, TUE selects an SUE $n\in N\left(t\right)$ and offloads its computing task. Assume that $x_{t}$( bits) represents the data size generated by the task transferred from TUE to SUE in time period T, $h_{t,n}^{\left(u\right)},n\in N\left(t\right)$ represents the wireless channel status of the uplink between TUE and SUE, and $I_{t,n}^{\left(u\right)},n\in N\left(t\right)$ represents the interference of SUE n. During the offload process of each computing task, the state of the wireless channel is kept static. We assume that transmission power is P. W and $\sigma^{2}$ represent the channel bandwidth and noise power, respectively; according to Shannon Hartley’s theorem, the uplink transmission rate of TUE and SUE can be expressed as:

(1) $$r_{t,n}^{\left(u\right)}=Wlog_{2}\left(1+\frac{P\cdot h_{t,n}^{\left(u\right)}}{\sigma^{2}+I_{t,n}^{\left(u\right)}}\right)$$

The transmission delay of offloading tasks to SEU n at time t is expressed as:

(2) $$d_{up}\left(t,n\right)=\frac{x_{t}}{r_{t,n}^{\left(u\right)}}$$

Task processing: Task processing begins after the selected SUE receives the task from TUE. In time period T, the total workload generated by tasks offloading is $x_{t\;}w_{t}$, where $w_{t}$ represents the computational intensity that the CPU cycle requires to process one bit of input data—it depends mainly on the nature of the application.

The maximum CPU resource within each computing cycle $F_{n}{}_{\;}$ represents the computing power of SUE n, and $f_{t,n}\in \;\left(0,F_{n}\right)$ is the CPU resource allocated to TUE at time t. SUE can process multiple computing tasks in parallel and dynamically adjust the CPU resource using dynamic voltage and frequency scaling (DVFS) technology [26].

Assuming that each computation task can be completed within a specific time period and every time slot of T is equal, the tasks with a larger workload can be divided into several sub-tasks, which are offloaded to the SUE n for a period of time and further processed. Then, the computing delay can be expressed as:

(3) $$d_{com}\left(t,n\right)=\frac{x_{t}w_{t}}{f_{t,n}}$$

which can be written as:

(4) $$d_{com}\left(t,n\right)=\frac{x_{t}w_{t}}{f_{t,n}}\frac{1}{\frac{\gamma_{t}}{x_{t}}-\sum_{n\in N\left(t\right)}k_{t,n}\lambda_{t,n}}\;$$

After the task processing is complete, the selected SUE n sends the result back to the TUE. Due to the very small amount of result data, the delay of result return is not taken into account here.

Then, at time t, when offloading tasks to SEU n, the total delay is:

(5) $$d_{sum}\left(t,n\right)=d_{up}\left(t,n\right)+d_{com}\left(t,n\right)$$

Our goal is to minimize the total offload delay by guiding the decisions of TUEs and maximize the computation resources of SUE. In each time slot of time period T, the workload offloading problem is formulated as follows:

(6) $$P1:\underset{\alpha_{1}\ldots \alpha_{T}}{min}\frac{1}{T}\overset{T}{\underset{t=1}{\sum }}d_{sum}\left(t,\alpha_{t}\right)$$

Here,$\;\alpha_{t}$ is an optimization variable, indicating that SUE’s index is selected in time period t.

Acquire state information: The device state information on the TUE side and the SUE side is obtained, including the data size of input data generated by each task $x_{t}$, and the computational intensity $\omega_{t}$. The uplink transmission rate is $r_{t,n}^{\left(u\right)}$, and $f_{t,n}$ represents the CPU resource assigned to each task calculation. The total delay can be calculated assuming that all of these states are fully known before each task is offloaded, and the optimization problem P1 above can be expressed as:

(7) $$P2:\alpha_{t}=\underset{n\in N\left(t\right)}{min}\;d_{sum}\left(t,n\right)$$

However, due to the temporal and spatial dynamics of user devices, data transmission rates change rapidly and are difficult to predict. Meanwhile, the exchange of the status information between the TUE and candidate SUEs results in signaling overhead, which is high. As a result, the TUE may lack information about the status of the SUE and may not be able to provide minimum latency for manufacturing decisions on its own.

Learning while offloading: To solve the above-mentioned problems, a learning method during offloading is introduced. TUE can observe and learn during offloading computing tasks and select candidate SUEs based on past offloading experience, with no need to obtain the exact transfer rate and CPU resource. When generating tasks through the same type of application, let the input data size be time-varying, and the computational intensity $w_{t}$ is constant. Assuming that $D_{k}$ is the maximum allowable computation delay for task requests of TUE and $C_{n}$ is the maximum computation capacity of SUE n, which represents the size of task data that SUE n can simultaneously process at time t, each task request can only be offloaded into one SUE. Then, the total delay can be written as:

(8) $$d_{sum}\left(t,n\right)=x_{t}\left(\frac{1}{r_{t,n}^{\left(u\right)}}+\frac{w_{t}}{f_{t,n}}\right)$$

Then, we have:

(9) $$P3:\underset{n\in N\left(t\right)}{min}\;\overset{T}{\underset{t=1}{\sum }}k_{t,n}(\frac{x_{t}}{r_{t,n}^{\left(u\right)}}+\frac{1}{\frac{\gamma_{t}}{x_{t}}-\sum_{n\in N\left(t\right)}k_{t,n}\lambda_{t,n}}\;)$$

(10) $$s.t.\;\;\overset{T}{\underset{t=1}{\sum }}\gamma_{t}\;\leq C_{n},\forall \;n\epsilon N\left(t\right)$$

(11) $$\;\;\;\overset{T}{\underset{t=1}{\sum }}d_{sum}\left(t,a_{t}\right)<D_{k}$$

(12) $$\;\;\;\;\;\;\;\;\;\overset{T}{\underset{t=1}{\sum }}k_{t,n}=1\;,\forall \;n\epsilon N\left(t\right)$$

(13) $$\;\;\;\;\;\;\;\;\;\;\;\;\;\;\;\;\;\;\;\;\;\;\;\;\;\;\frac{\gamma_{t,n}}{x_{t}}-\overset{T}{\underset{t=1}{\sum }}k_{t,n}\lambda_{t,n}>0,\forall \;n\epsilon N\left(t\right)$$

(14) $$k_{t,n}\in \left\{0,\;1\right\}$$

Constraint (10) indicates that the task assigned to SUE at time t cannot exceed its computational capacity. Constraint (11) ensures that the total computation delay of the entire iteration cycle is less than the maximum allowable computation delay. Constraint (12) means that each TUE selects an SUE. The average task arrival rate for SUE cannot be greater than its average service rate, as shown in constraint (13); otherwise, the offloading request will accumulate indefinitely.

The decision problem P3 can be transformed into a partition problem, which is known as an NP-complete problem. Since the decision problem for P3 is NP-complete, P3 is NP-hard [27].

### 3.3. The SAda Algorithm

The task offloading problem P3 can be solved according to Multi-Armed Bandit (MAB) [28] theory, which requires online sequential decision making. The TUE is the decision maker of task offloading, and it requires constant learning to estimate losses in order to minimize the sum of cumulative losses. Based on the problem, we propose a self-adaptive learning task offloading algorithm (SAda) for MEC systems, which can sense both the size of the input data and the occurrence of the user’s device and enables TUE to learn from the offloading experience of the candidate SUE.

In Algorithm 1, the parameter β is constant,$\;\forall t,w_{t}=w_{0}$, and $k_{t,n}$ denotes the count of tasks offloaded into SUE n at time t.$\;t_{n}$ represents the occurrence time of SUE n; Lines 7–14 are the main cycle of the learning process, which is inspired by the Upper-Confidence-Bound (UCB) algorithm [29] and MAB mentioned above. At the beginning of each iteration, before offload tasks and calculation $x_{t}$, the TUE obtains the input data size $x_{t}$.
**Algorithm 1:** The SAda Algorithm.1:
Initialization Parameter:
$x_{t},\alpha_{0},\omega_{0}$
and
$\sigma^{2}$
2:**for** t in T **do**
3: Get the location of IoT devices and select the suitable devices as the candidates, update $N\left(t\right)$.
4:
 **if** none of SUE
$n\in N\left(t\right)$
has connected to TUE **then**
5:
  Connect to SUE n once.
6:
  Update
$u_{t,n},k_{t,n},t_{n}$
.
7:
 **else**
8:
  Observe
$x_{t}$
.
9:
  **for** each device
$n\in N\left(t\right)$
**do**
10:
   Calculate the efficiency function
$u_{t,n}$
.
11:
   Find device
$\alpha_{t}$
,where
$\alpha_{t}=arg\underset{n\in N\left(t\right)}{\min}u_{t,n}$
.
12:
  Update
$d_{sum}\left(t,\alpha_{t}\right)$
.
13:
  Update
$\bar{u}\left(t,\alpha_{t}\;\right)=\frac{{\bar{u}}_{t-1,\alpha_{t}}k_{t-1,\alpha_{t}+d_{sum}\left(t,\alpha_{t}\right)}}{k_{t-1,\alpha_{t}}+1}\;\;\;\;\;\;\;\;\;\;\;\;\;\;\;\;\;\;\;\;\;\;\;\;\;\;\;\;\;\;\;\;\;\;\;\;\;\;\;\;\;\;\;\;\;\;\;\;\;\;\;\;\;\;\;\;\;\;\;\;\;\;\left(15\right)$
14:
  Update
$k_{t,\alpha_{t}}=k_{t-1,\alpha_{t}+1}$
15:
  **end for**
16:
 **end if**
17:**end for**

The padding function defined in Equation (15) is used to evaluate the service capacity of each SUE, which is derived from the empirical bit offloading delay $\;\bar{u}\left(t,n\right)$. Specifically, it takes into account both the input data size and the time of occurrence of each SUE. Given the limited computing resources of users’ devices, it is necessary to balance SUE exploration and task processing in the process of offloading task learning. Reasonable allocation of computing resources can ensure that they both obtain the best improvement under the limited computing resources and also adapt to the dynamic MEC environment.

The SAda algorithm has the following two prominent advantages:

Service capacity assessment: The algorithm can estimate the possible delay impact of offloading the task to the SUE by combining the size of the input data with the remaining computational resources of the SUE. The input data size can affect the computation latency of different SUEs.

Occurrence awareness: Considering the temporal and spatial dynamics of an SUE, a solution is set for it. Specifically, for any SUE that is detected for the first time,$\;k_{t-1,n}$ is inversely proportional to $\sqrt{\frac{ln\left(t-t_{n}\right)}{k_{t-1,n}}}$. Smaller padding functions mean that SAda takes advantage of more learning information.

The whole task processing time is defined as T, and there are C epochs in time T, $c_{1},\;c_{2}\ldots$. The unit learning error *e* represents the difference in the delay of offloading one bit of the task during the learning process. $\hat{u}$ represents the optimal bit offloading delay in each epoch. Then, the learning error in the whole learning process can be expressed as:

(16) $$E_{T}=\overset{C}{\underset{c1}{\sum }}\overset{T}{\underset{t=1}{\sum }}x_{t}\left(u\left(t,n\right)-\hat{u}\right)$$

**Lemma** **1.** *After a finite number of iterations, the SAda algorithm will terminate after completing the flexible assignment of tasks.*

**Proof.**  Let $\tau =\left|N\left(1\right)\right|=m,\;\tau >0.$ Initially,$\;N\left(1\right)$ represents the set of SUEs that have not been assigned tasks. Then, the algorithm selects the appropriate SUE $a_{t}$ for each iteration with minimum bit delay and offloads the task on the TUE to it. With the execution of the algorithm, τ decreases gradually and finally approaches zero; $N\left(1\right)=\emptyset$ after a finite number of iterations. □

As shown in the SAda algorithm, the complexity of calculating the efficiency function is $\left|Z\right|$ in Line 8; the minimum seeking problem in Line 10 has a complexity of $\left|Z\right|$. $O\left(1\right)$ is the complexity of Line 12–13. Therefore, the total algorithm complexity in each time period is $O\left(Z\right)$. Assuming that the number of task requests is I during the entire offloading process in the MEC system, the total algorithm complexity is $O\left(IZ\right),$ which is relatively lower than the existing algorithm.

**Lemma** **2.** *When each TUE finds a suitable SUE, X is determined and P3 becomes a convex optimization problem.*

**Proof.**  To prove the problem, let $y=k_{tn}(\;\frac{x_{t}}{r_{t,n}^{\left(u\right)}}+\frac{1}{\frac{\gamma_{t,n}}{x_{t}}-\sum_{t\in T}k_{tn}\lambda_{tn}})$, and take the second derivative with respect to $\gamma_{t}$; then, we have $\frac{\partial^{2}y}{\partial^{2}\gamma_{t,n}}=\underset{t\in T}{\sum }2k_{tn}x_{t}^{-2}{\left(\frac{\gamma_{t,n}}{x_{t}}-\underset{t\in T}{\sum }k_{tn}\lambda_{tn}\right)}^{-3}$. The function y is convex because of the positive definite Hesse matrix $Y=\frac{\partial^{2}y}{\partial^{2}\gamma_{t,n}}$. □

By solving the KKT condition of P3 [30], the optimal solution of P3 can be acquired as a convex problem. As a result, the optimal task offloading scheme with TUE to SUE obtains the optimal solution with the lowest response time.

## 4. Simulations and Discussion

In this section, the average delay performance of user devices is evaluated under different moving speeds of the proposed SAda algorithm and implemented algorithm simulations. The influence of key parameter changes on the time delay in the composite scene is evaluated to simulate the real street scene. The parameters used in this simulation are shown in Table 2. The Wi-Fi transmission, which can access wireless signals at any time and has strong mobility, is considered as the main transmission technology in this paper.

The simulation environment consists of 10 SUEs and 1 TUE. A 12 km segment of road in Beijing is downloaded from Open Street Map and used in our simulations. SUEs are randomly distributed around the TUE and move in the same direction [24]. The emergence and leaving time of SUEs are shown in Table 3. In the T=3000 time periods, the whole iteration cycle is divided into three periods and each epoch lasts 1000 time periods. The communication ranges of the TUE and SUEs are set to $\left[0,300\right]$, channel bandwidth W=10 MHz. According to the inverse power law $h_{t,n}^{u}=A_{0}l^{-2}$, the wireless channel state is modeled with $A_{0}=-17.8\;\mathrm{dB}$, and l is the distance between TUE and SUE [31]. The computational intensity $w_{0}=1000\;\mathrm{Cycle}/\mathrm{bit}$ represents the CPU cycles required to process one bit of input data, and it mainly depends on the nature of the application. The input data size $x_{t}$ is generated uniformly between $\left[0.1,\;1\right]$, and the transmission power P=0.1 W, which mainly depends on the transmission distance l. According to the Shannon–Hartley theorem, the noise power $\sigma^{2}=10^{-13}\;$W.

To evaluate the performance of the proposed algorithm, SAda, we compare it with three existing algorithms. (1) The Upper-Confidence-Bound (UCB) algorithm [29] is neither occurrence awareness nor input data size sensing. (2) The volatile UCB (VUCB) algorithm [32] can sense an SUE’s presence. (3) The adaptive UCB (AdaUCB) algorithm [33] has the ability to sense the size of input data. 

**Figure 2:**
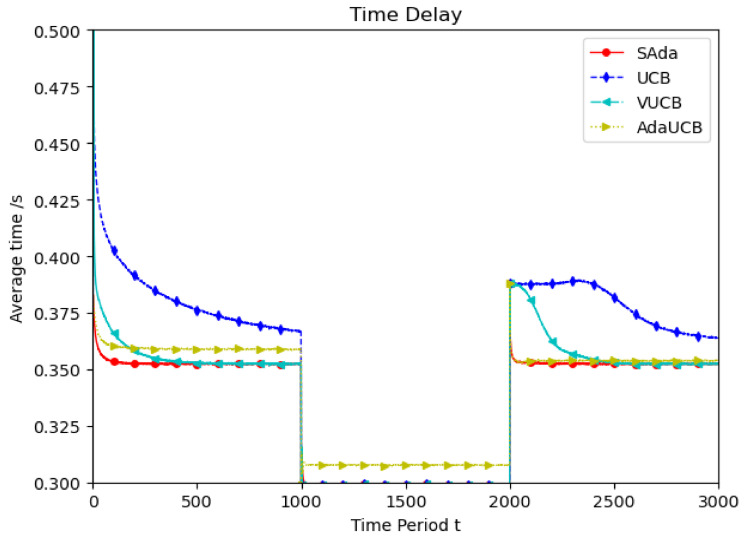
Average delay comparison between SAda and three other existing algorithms at TUE speeds of 60 km/h.

Considering the spatiotemporal dynamics of the TUE, we simulate it at low speed, high speed and random speed. As shown in Figure 2, TUEs with a faster moving speed of 60 km/h are considered as the experimental object. In the first epoch, UCB and VUCB have the same approach rate, and the optimal average delay is higher. SAda and AdaUCB have a lower delay and approach rate than UCB and VUCB. Further, SAda has the minimum average delay and optimal delay approach rate. During the second period, due to the emergence of more SUEs in the system, the task requirements of TUE can be processed more fully and quickly, so that the optimal delay is reduced. With the departure of SUEs, the system’s task processing delay increases. SAda also has the lowest approach rate and the fastest optimal delay approach rate compared to the other algorithms in the third epoch. It is obvious that, throughout all the epochs, SAda has the optimal delay and approach rate.

**Figure 3:**
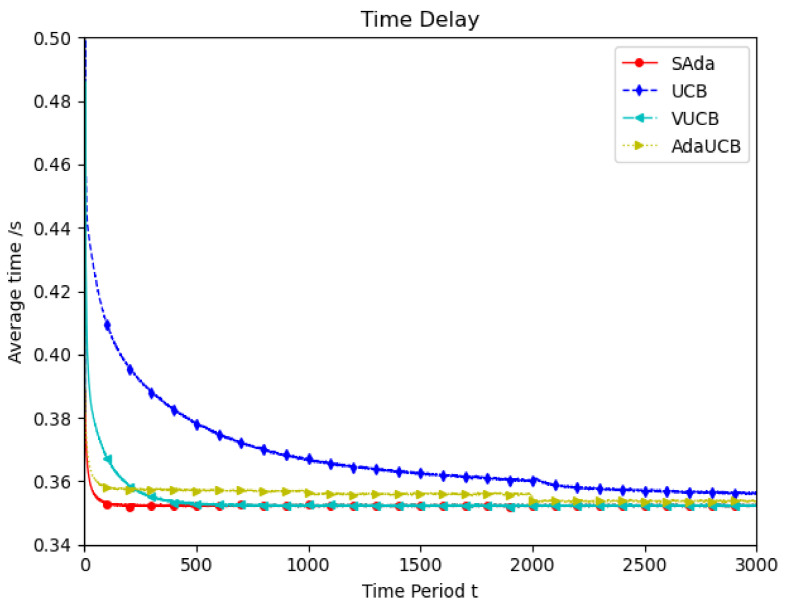
Average delay comparison between SAda and three other existing algorithms at TUE speeds of 10 km/h.

**Figure 4:**
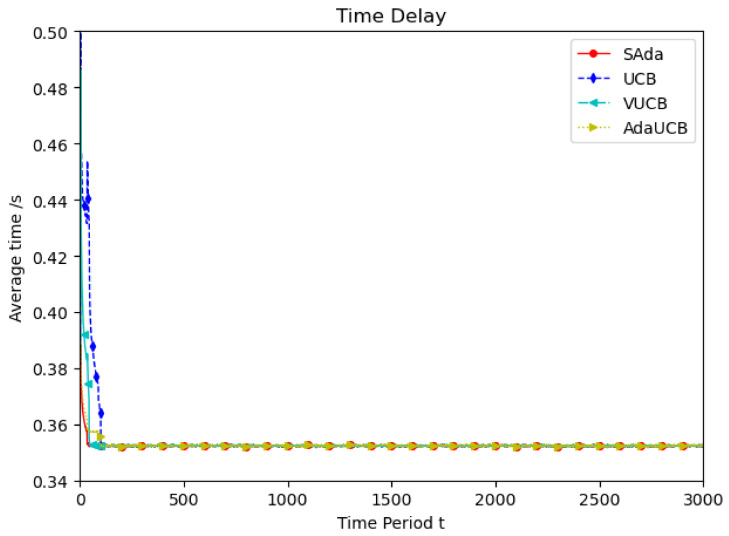
Average delay comparison between SAda and three other existing algorithms at random TUE speed.

Figure 3 shows the simulation of user devices with lower mobile speeds, which considers the mobility characteristic of user devices in the real world. Throughout the entire period, compared with the other three algorithms, SAda has a faster optimal delay convergence speed and optimal average delay when the speed of TUEs is 10 km/h. Compared with the VUCB and AdaUCB algorithms, SAda can improve the convergence of optimal delay by 80% to 95%. In order to adapt to the real environment, the simulation is performed at random speed and the result is shown in Figure 4. As mobile devices move at a variety of speeds, SAda still has the best optimal delay convergence rate, response delay and a significant performance improvement. Because SAda has both the input perception and consciousness perception function, it can find SUEs for TUE more quickly and select an appropriate SUE for task offloading.

**Figure 5:**
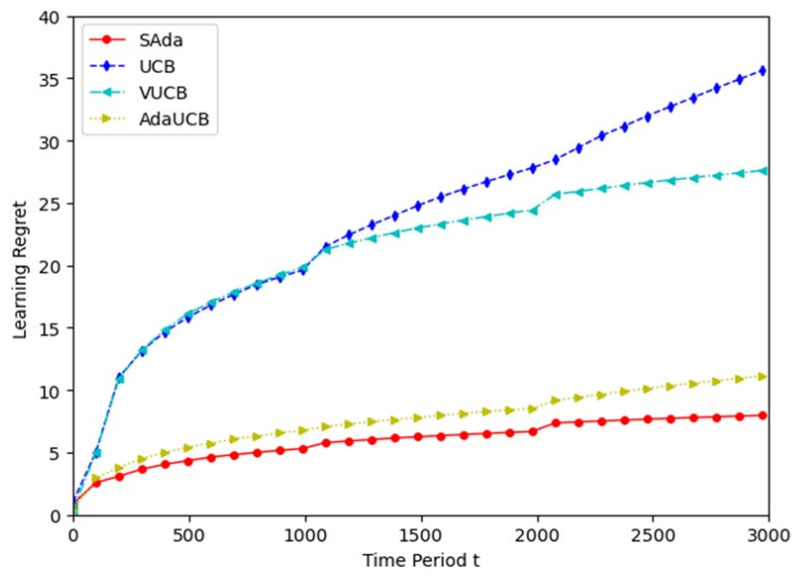
Comparison diagram of algorithm learning error.

Figure 5 evaluates the learning errors of the three algorithms. The SAda algorithm has the lowest learning error in the whole period, and the learning error during the process of task offloading can be increased by around 20%, 70% and 75%, respectively, compared with the three existing algorithms. The learning errors of VUCB and AdaUCB are smaller than that of UCB, which indicates that input perception and consciousness perception enhance the adaptability of the MEC system, and considering both can further improve the performance of the system. Through extensive simulation, it is proven that SAda has lower task processing delay and learning error, with better performance when compared with the other three algorithms.

## 5. Conclusions

In this paper, we have investigated the problem of offload allocation in MEC systems, and a novel self-adaptive learning task offloading algorithm, SAda, for MEC systems is proposed to minimize the task processing delays while considering the spatiotemporal dynamics of mobile devices. On the basis of the original MAB algorithm, the perception function and the offloading learning mechanism have been considered to improve the speed of the task transmission processing. Through extensive simulation, it is proven that SAda has a lower delay rate and better performance compared with the existing algorithms. Compared with the VUCB and AdaUCB algorithms, SAda can improve the convergence of optimal delay by 80% to 95%, and the learning error during the process of task offloading can be increased by 20%, 70% and 75%, respectively. 

In the future, the task offload situations when there are multiple TUEs will be considered in order to better match the reality. Moreover, a faster learning network can be used for this work, and more complex task offloading between crossings and cloudlets attached to the roadside should be considered in order to further optimize the delay performance.

## Figures and Tables

**Table 1:** List of symbols.

Symbol	Definition
$N\left(t\right)$	Set of candidate SUEs
B	Set of computational epochs
$x_{t}$	Data size generated by tasks transferred from SUE to SUE at time t
$\alpha_{t}$	Index of the selected SUE in time period t
$h_{t,n}^{\left(u\right)}$	Uplink wireless channel state of TUE and SUE n at time t
$I_{t,n}^{\left(u\right)}$	Uplink interference of SUE n at time t
$r_{t,n}^{\left(u\right)}$	Uplink transfer rates for TUE and SUE n
$\lambda_{t,n}$	Average task arrival rate of SUE n
$d_{up}$	Transmission delay of uploading the task to SEU n at time t
$d_{com}$	Computing delay of the task transferred to SEU n at time t
$F_{n}$	Maximum CPU resource
$f_{t,n}$	CPU resource assigned to the TUE at time t
$D_{k}$	The maximum allowable computation delay for task requests of TUE
$C_{n}$	The maximum computation capacity of SUE n
W	Channel bandwidth
P	Transmission power
$\sigma^{2}$	Noise power
$u_{t,n}$	Delay of offloading a bit of input data to SUE n at time t
$\gamma_{t}$	Computing capacity of the selected SUE at time t
$k_{t,n}$	Binary index of SUE n selected by TUE at time t

**Table 2:** Parameters used in the simulation.

T	W	$A_{0}$	$w_{0}$	P	$\sigma^{2}$
3000 s	10 MHz	−17.8 dB	1000 Cycle/bit	0.1 W	$10^{-13}$ W

**Table 3:** SUE discovery and disappearance epochs.

SUEs	1	2	3	4	5	6	7	8	9	10
**Epoch1**	√	√	√	√	√	—	—	—	√	—
**Epoch2**	√	√	√	√	—	√	√	—	√	√
**Epoch3**	—	√	—	√	√	—	√	√	—	√

## Data Availability

The data presented in this study are available on request from the corresponding author. The data are not publicly available due to restrictions of privacy and morality.
